# Addiction service changes due to COVID-19

**DOI:** 10.1192/bjo.2021.914

**Published:** 2021-06-18

**Authors:** Shumaila Shahbaz, Zeeshan Hashmani, Soraya Mayet

**Affiliations:** 1 Humber Teaching Foundation Trust; 2 Tees Esk and Wear Valley NHS Foundation Trust

## Abstract

**Aims:**

Addictions services had to respond rapidly to reduce COVID-19 transmission to protect patients and staff. Patients with opioid dependence are particularly vulnerable, with high risks. Our community addiction service changed practice in line with COVID-19 guidelines. For patients with opioid dependence; face-to-face contacts were initially reduce and mainly for new starts, restarts and non-attenders. Prescribing changes were completed on an individually risk assessed basis to reduce attendance at the chemist, specifically to reduce transmission, keep patients in treatment and to ensure chemists could continue to function. We document some of the service changes during the COVID-19 lockdown.

**Method:**

Service evaluation had approval from Humber Teaching NHS Foundation Trust. Data retrieved on one Hub of a community addictions service in North England, UK. Patients prescribed opioid substitution treatment for opioid dependence were assessed, with data retrieval through electronic healthcare records. Data were analysed by Microsoft Excel anonymously.

**Result:**

In lockdown (March 2020 to June 2020), we identified 112 patients with opioid dependence prescribed opioid substitution (OST) with methadone or buprenorphine at the Hub. All white British, mean 42 years, most male (75%) and prescribed methadone (78%). Ten were new starts and 8 restarts to OST. Attendance rates did not change: 91% before and 92% during lockdown. Appointment format changed from predominantly face-to-face (92%) to telephone (99%). Most patients (91%;n = 88) were offered take-home naloxone and overdose prevention training of which 14 refused. Supervision days at the chemist for OST reduced significantly from 75% collecting daily at the chemist, reducing to 20% during lockdown. Five patients were shielding and 7 had covid-related symptoms. There was one death during lockdown which was not attributed to covid or overdose.

**Conclusion:**

The addictions service continued to be open and work proactively throughout lockdown, seeing new patients and continuing treatment interventions safely. Major changes were made in line with COVID-19 guidelines, to respond to the threat of transmission. Our service was flexible and able to adapt quickly to remote working. We maintained excellent attendance rates despite changes to the format of consultations. There were no related incidents e.g. overdoses linked to prescribed medications, despite a reduction in supervision, and therefore patients having extra medications. This important finding may be related to the individual risk assessments that we conducted before making changing to prescribing. This was supported by most patients were receiving naloxone to prevent overdoses. Some of the changes, such as telephone consultations, may be beneficial to continue post COVID-19.

